# Knockout of *BnaX.SGT.a* caused significant sinapine reduction in transgene-free rapeseed mutants generated by protoplast-based CRISPR RNP editing

**DOI:** 10.3389/fpls.2024.1526941

**Published:** 2025-01-07

**Authors:** Oliver Moss, Xueyuan Li, Eu Sheng Wang, Selvaraju Kanagarajan, Rui Guan, Emelie Ivarson, Li-Hua Zhu

**Affiliations:** Department of Plant Breeding, Swedish University of Agricultural Sciences, Lomma, Sweden

**Keywords:** rapeseed and canola, genome editing by CRISPR-Cas, protoplast-based CRISPR RNP, DNA-free, transgene-free mutants, seedcake quality, antinutritional factor or compound

## Abstract

Rapeseed (*Brassica napus* L.) is known for its high-quality seed oil and protein content. However, its use in animal feed is restricted due to antinutritional factors present in the seedcake, with sinapine being one of the main compounds that reduces palatability. Attempts to develop rapeseed germplasm with lower sinapine levels through traditional breeding methods have shown limited progress. Genetic transformation methods could create new genotypes with reduced sinapine levels by silencing key genes involved in sinapine biosynthesis, though these methods often result in transgenic or genetically modified plants. The recent development of CRISPR-Cas technology provides a precise and efficient approach to crop improvement, with the potential to generate transgene-free mutants. In this study, we targeted the *BnaX.SGT.a* genes for knockout using CRISPR-Cas editing. By utilizing our newly established protoplast regeneration and transfection protocol for rapeseed, we demonstrated that DNA-free CRISPR editing via protoplast-based ribonucleoprotein (RNP) delivery was highly effective. We achieved successful knockout of the *BnaX.SGT.a* paralogues, with an average mutation efficiency of over 30%. Sequencing results revealed a variety of mutation types, from 1 bp insertions to 10 bp deletions, with most mutants exhibiting frameshift mutations that led to premature stop codons. The mutants displayed no visible phenotypic differences in growth patterns or flowering compared to the wild type. Importantly, sinapine content was significantly reduced in all T_2_ generation mutants analysed, while seed weight remained comparable between mutants and the wild type.

## Introduction

Rapeseed (*Brassica napus* L.) is the third largest source of vegetable oil in the world, after palm and soybean, and it is the main oil crop grown in Europe (Shahbandeh, 2024). The seedcake remaining after oil extraction is rich in high-quality protein with a favourable amino acid profile (Cheng et al., 2022). This gives rapeseed significant potential as a plant protein source for feed and food applications, a role which is primarily fulfilled by imported soybean meal in Europe at present. Currently, the use of rapeseed seedcake is limited in animal feed due to the presence of antinutritional factors. Sinapine is one such factor; it reduces palatability, inhibits nutrient absorption, and causes the eggs of certain species of chicken to have a fishy taste (Kozlowska et al., 1990; Qiao et al., 2008; Ward et al., 2009). In rapeseed, sinapine functions as a storage compound for choline, while also offering antioxidant properties and protection against UV-B radiation (Yates et al., 2019; Sheahan, 1996).

Reducing sinapine levels in rapeseed seeds could transform rapeseed meal from a low-value by-product into a high value protein source. This shift is particularly important in the face of a changing climate, as extreme weather events put increasing pressure on agriculture. With rising temperatures, new areas are becoming suitable for certain pests and pathogens, creating challenges for crops that are not adapted to these threats. Additionally, unpredictable weather patterns can negatively affect crop yields, and the rapid speed of environmental changes outpaces the ability of plants to naturally adapt (Jacobs et al., 2024). Given these challenges, maximizing the utility of the side streams of agricultural crops is crucial. For rapeseed, this could mean expanding its value beyond oil production to include high-quality protein, thereby adding economic value and enhancing sustainability by utilising the entire crop—including the seedcake, a current side stream of rapeseed oil production.

There are several methods for inducing mutations in target genes to improve traits, each with distinct advantages and limitations. Spontaneous mutations arise naturally without external intervention, maintaining the organism’s native genetic background. However, their unpredictable nature and minor phenotypic effects often necessitate extensive screening to identify beneficial mutations. TILLING combines mutagenesis with targeted screening, enabling the identification of mutations in specific genes. This method is highly scalable, allowing the simultaneous screening of many individuals. Nevertheless, TILLING relies on random mutagenesis, which can produce nonspecific or off-target mutations that may be harmful or impair overall plant viability, and is very labour intensive. RNAi technology enables the silencing of specific genes, resulting in desired phenotypic traits. However, its application leads to genetically modified organisms (GMOs), which can face regulatory and public acceptance challenges.

The modern site-directed mutagenesis technology, such as CRISPR-Cas gene editing, provides promising solutions for modern plant breeding challenges. This technology enables the rapid and precise introduction of mutations in the host genome without integrating foreign DNA, and it has a low risk of off-target effects (DeWitt et al., 2017). Additionally, recent EU proposals suggest a potential relaxation of regulations on new genomic technologies (NGTs), including CRISPR-Cas-based approaches (Dionglay, 2024). Consequently, CRISPR-Cas-mediated mutation breeding addresses the limitations associated with traditional breeding, TILLING, and genetic engineering approaches, genetic engineering approaches, and TILLING, providing a more precise, efficient, and flexible approach for gene knockout. The estimated need to double the rate of genetic improvement to meet changing environmental demands aligns well with the potential of CRISPR-Cas mutagenesis (Voss-Fels et al., 2019). By reducing breeding cycles, CRISPR-Cas can produce new lines in half the time required by conventional breeding methods (May et al., 2023). This relationship highlights the critical role of CRISPR-Cas technology in modern plant breeding for meeting the urgent challenges faced in food supply from a growing global population in a changing climate.

Protoplast-based gene editing approaches are an efficient tool for achieving transgene-free gene editing, as they allow the direct delivery of CRISPR components across permeable cell membranes. This can be accomplished either by transiently expressing CRISPR components from plasmids or by introducing a ribonucleoprotein (RNP) complex composed of the Cas9 nuclease and a single guide RNA (sgRNA). Although both methods can generate transgene-free mutants, the RNP-based approach offers distinct advantages over plasmid-based methods. RNPs enable DNA-free editing, eliminating the need for plasmid vector preparation and removing concerns about plasmid DNA integration into the host genome. Additionally, RNPs enhance editing efficiency and reduce the likelihood of off-target mutations (Zhang et al., 2021; DeWitt et al., 2017). The primary challenge of protoplast-based methods lies in the difficulty of protoplast regeneration, which can vary significantly depending on the plant species or even the specific genotype. Recently, Li et al. (2021) developed an efficient protocol for regenerating rapeseed protoplasts and demonstrated the successful delivery of CRISPR plasmids to create mutant lines.

SGT (UGT84A9) (UDP-glucose:sinapate glucosyltransferase) is identified as a key enzyme in sinapine biosynthesis (Milkowski et al., 2004). There are two *SGT* genes in the *B. napus* genome, *BnaX.SGT.a* and *BnaX.SGT.b*, each located on both the A-genome and the C-genome, resulting in four loci: *BnaA.SGT.a*, *BnaA.SGT.b*, *BnaC.SGT.a*, and *BnaC.SGT.b* (Mittasch et al., 2010) *BnaX.SGT.a* is predominately expressed in developing seeds, while *BnaX.SGT.b* has negligible expression, apart from in flowers (Mittasch et al., 2010). Silencing *BnaX.SGT.a* through RNA interference (RNAi) has successfully reduced sinapine accumulation in rapeseed seeds (Hüsken et al., 2005; Wolfram et al., 2010). Furthermore, *BnaX.SGT.a* knockout lines generated via TILLING-based EMS mutagenesis demonstrate the potential for reducing sinapine accumulation through mutagenesis (Emrani et al., 2015). These findings underscore the role of *BnaX.SGT.a* in sinapine accumulation. Importantly, research investigating the effects of *SGT* silencing has shown no adverse impacts on agricultural traits, including yield and oil content, as well as seed development, germination, or sensitivity to UV-B radiation (Hüsken et al., 2005; Wolfram et al., 2010; Hettwer et al., 2016). In this study, we aimed to target the two *BnaX.SGT.a* paralogues for reducing the sinapine levels in rapeseed seed of transgene-free mutants by protoplast-based CRISPR RNP editing.

## Materials and methods

### Plant material

The seeds used in this study were spring rapeseed (*B. napus* L.) cv. Kumily, a doubled haploid, provided by Lantmännen, Sweden.

### Seed sterilisation

Seeds were surface sterilized by gentle shaking in 70% ethanol for 15 minutes, followed by gentle shaking in 20% kitchen bleach for 15 minutes. The seeds were then washed in sterile water four times.

### *In vitro* seedling growth and growth conditions

Sterilized seeds were grown on the germination medium in single-use sterile plastic boxes. The germination medium contained half strength Murashige & Skoog (MS), 10 g l^−1^ sucrose, 7 g l^−1^ Bacto agar at pH 5.7. The boxes were placed in a climate chamber, which had a 16 hr photoperiod, with a light intensity of 40 μmol m^−2^ s^−1^ (cool white fluorescent tubes). The temperatures were 23°C/18°C for day and night respectively.

### Sequencing of *BnaX.SGT* paralogues

For sequencing the *BnaX.SGT.a* and *BnaX.SGT.b* paralogues, DNA was extracted from the leaves of rapeseed cv. Kumily using the GeneJet Plant Genomic DNA Purification Mini Kit (Thermo Fisher Scientific, USA). PCR was performed using primers (**Table 1**), targeting all four paralogues of the two genes, designed using the sequences for *BnaX.SGT.a* (UGT84A9-1) and *BnaX.SGT.b* (UGT84A9-2) from NCBI. The PCR product was purified using NucleoSpin Gel and PCR Clean-up, Mini kit (Macherey-Nagel, Germany) according to the manufacturer’s instructions. The purified PCR product was then cloned into the pJET1.2/blunt vector using the CloneJET PCR Cloning Kit (Thermo Scientific, USA). Transformation was conducted using Stellar™ Competent Cells (Takara Bio, Japan) to propagate the recombinant plasmid. Plasmid DNA was subsequently purified from bacterial cultures with the NucleoSpin Plasmid Mini Kit (Macherey-Nagel), yielding high-purity plasmid DNA suitable for downstream applications. The recombinant plasmids of 16 colonies were then sent to Eurofins (Germany) for Sanger sequencing of the genes.

**Table 1:** CRISPR target sequences and primers for gene sequencing and target site amplicon sequencing.

CRISPR target sequence (CTS) or primer	Sequence *	Purpose
CTS1 (sgRNA1) CTS2 (sgRNA2) Forward primer Reverse primer Forward primer Reverse primer	GGACCCAGAGAACAGCACAG**GGG** ATTTTGTAAAGCGGTCCGAG**CGG** AGCACACAGAAGAGAACCCC TCAGGATTTGCAGAAAACAAACA GCTGGTCGGACAACAAGAGA CTGCGAGTCTAACCACTCCA	Gene knockout	Gene knockout	Sanger gene sequencing	Sanger gene sequencing	Target site amplicon sequencing	Target site amplicon sequencing

*The sequence orientation of the CTSs is 3**’**-5**’** with the PAM sites in bold.

### Design of sgRNAs

Geneious Prime 2024.0.5 was used to predict sgRNA sites, and its off-target checker tool was employed to assess potential off-target effects. Additionally, the online tool Cas-OFFinder (Bae et al., 2014) was used to further evaluate off-target sites (available at http://www.rgenome.net/cas-offinder/). Two sgRNAs (sgRNA1 at bp 425-442 and sgRNA2 at bp 735-758 of the gene) were selected based on high activity scores and absence of off-target effects, targeting on conserved functional domains as well as location in regions of high sequence homology. The two sgRNAs (**Table 1**) meeting these criteria most effectively were chosen for this study.

### Protoplast isolation, transfection and plant regeneration

Leaves from 18-21 day old seedlings were used for protoplast isolation using the method described by Li et al. (2021). Approximately 120,000 isolated and washed protoplasts were re-suspended in 200 µl freshly prepared MMG solution (0.5 M mannitol, 15 mM MgCl_2_, 4 mM MES) in a 2 mL Eppendorf tube for transfection. The solution was mixed with 20 µl RNP complex solution (4 µl gRNA (0.1 nmol/µl), 4 µl Cas9 (5 µg/µl), and 12 µl H_2_O), and 220 µl freshly prepared PEG-calcium solution (40% (w/v) PEG 4000, 0.5 M mannitol, 0.1 M CaCl_2_). The reaction was stopped after 6 min by addition of 1.5 ml W5 and gentle mixing by inversion of the tubes, followed by centrifugation for 3 min at 100 g and immediate removal of the supernatant. Protoplasts were then embedded in alginate discs and cultured in 6-well microplates, as described by Li et al. (2021). The embedded protoplasts were cultured for shoot regeneration according to the optimized protocol described in the same study. The *in vitro* regenerated shoots or putative mutants were rooted on the rooting medium, as described by Li et al. (2021). Once the shoots formed roots, they were transferred to soil pots and grown in the biotron growth chambers, where growth conditions were 21°C/16°C (day/night), 16 h photoperiod with a light intensity of 250 µ mol m^−2^ s^−1^ and 60% humidity.

### Identification of mutant lines

Leaf tissue was taken from *in vitro* regenerated shoots of putative mutants and crushed with a pipette tip in Phire Dilution Buffer (Thermo Fisher Scientific, USA). The supernatant was used as a template for a PCR reaction using Phusion High-Fidelity PCR Master Mix (Thermo Fisher Scientific, USA) and gene specific primers (**Table 1**) to amplify the target region containing the sgRNA site. The PCR products were purified using GeneJET Gel Extraction and DNA Cleanup Micro Kit (Thermo Fisher Scientific, USA) and sent for Sanger sequencing (Azenta Life Sciences, USA).

The seeds harvested from T_0_ were sewn in pots and grown in the biotron. Seeds from T_1_ plants were analysed for sinapine content and the lines with lower sinapine contents were sown for obtaining T_2_ seeds. Two mutants from each T_2_ line, as well as two WT, were sequenced using amplicon sequencing in order to acquire accurate sequences from all targeted alleles, and to elucidate the type of mutations caused. Genomic DNA was extracted from plants using the GeneJet Plant Genomic DNA Purification Mini Kit (Thermo Fisher Scientific, USA), and PCR was performed using Illumina adapter-linked primers to amplify the target region, which was then purified using GeneJET Gel Extraction and DNA Cleanup Micro Kit (Thermo Fisher Scientific, USA). The samples were then sent for amplicon sequencing at Eurofins Genomics (Germany).

### Extraction of sinapine

Sinapine was extracted from defatted rapeseed meal according to the method described by Wang et al. (1998) with modifications. In summary, 150 mg of pooled seeds from each plant in each generation (detailed numbers of plants for each generation are shown in Figures in the result section) were frozen at -80°C and milled using a Retsch MM 400 steel ball mill (Fisher Scientific, USA) at 30 Hz for 2 minutes. Sinapine extraction was then performed on 50 mg aliquots with 3 replicates per sample. For the defatting of samples, 1 ml of heptane (analytical grade) was added to each tube and the tubes were vortexed for 10 seconds and allowed to stand for 30 minutes at room temperature. The tubes were centrifuged at 10 000 rpm for 3 minutes and the supernatant was discarded. The defatting procedure was repeated once more with the standing time reduced to 5 minutes and the pellet was dried to completion in a vacuum desiccator (Concentrator Plus, Eppendorf, Germany). Sinapine was extracted from the defatted meal pellet by the addition of 1 ml of 70% methanol to each tube. The samples were vortexed for 10 seconds and placed in ultrasonic bath for 5 minutes. The tubes were then placed in a preheated heat block set to 75°C for 30 minutes. The tubes were centrifuged at 10,000 rpm for 2 minutes and the supernatant containing the crude sinapine extract was transferred to a new 2 ml screw-capped tube. To ensure complete extraction, another 1 ml of 70% methanol was added to the remaining pellet and the tube was vortexed for 10 seconds and allowed to stand at room temperature for 5 minutes. The tubes were centrifuged again at 10,000 rpm for 2 minutes and the supernatant was transferred to and mixed with the earlier collect. The samples were kept at -80°C until HPLC analysis.

### HPLC analysis of sinapine

Samples were prepared for HPLC analysis by mixing 1 ml of the crude sinapine extract with 1 ml of eluent (10 mM sodium acetate, pH 4.0, with 13.5% acetonitrile). The mixture was centrifuged at 13,500 rpm for 5 minutes to pellet any residual particles, and 600 µl of the supernatant was transferred to an HPLC vial. Samples were analysed on an Agilent 1260 Series HPLC system, with separation of sinapine achieved using an Eclipse Plus C18, 3.0 x 100 mm, 3.5 µm column (Agilent, USA). A 6-minute isocratic elution was applied at a flow rate of 1 ml/min with a single eluent (10 mM sodium acetate, pH 4.0, with 13.5% acetonitrile). Sinapine was detected photometrically using a variable wavelength detector (VWD) at a signal wavelength of 330 nm and quantified based on its retention time with respect to a certified sinapine external standard (ChemFaces, China).

### Phenotypic observations

Apart from regular visual observations on growth, flowering, seed setting etc., mature plants were photographed at harvest using a mirrorless interchangeable-lens camera (MILC; X-T3, Fuji). 100-seed weight was measured using a microbalance (RE 1614, Sauter).

### Statistical analysis

Data for sinapine content and 100-seed weight were analysed with ANOVA and Tukey’s test, with the significance level set at *p* = 0.05 using the Minitab statistical program (Minitab, LLC (USA), version 21.4.2.

## Results

### Gene sequencing

The target genes were sequenced using Sanger sequencing with homology-based primers (**Table 1**). The sequencing results confirmed the presence of four *Bna.SGT* paralogues in rapeseed cv. Kumily. These paralogues fall into two distinct sequence types: *BnaX.SGT.a* and *BnaX.SGT.b*. Both sequence types are found in the A and C genomes of rapeseed, referred to as *BnaA.SGT.a*, *BnaA.SGT.b*, *BnaC.SGT.a*, and *BnaC.SGT.b*. The sequences of all *Bna.SGT* paralogues were obtained in cv. Kumily with the sequence-specific primers, which had 100% identity with the sequences from the NCBI database for all paralogues.

### Protoplast transfection and screening of regenerated plants

The RNP transfected protoplasts regenerated well *in vitro* with a large number of shoots formed. Fifty T_0_ shoots were screened for mutations on the target gene using Sanger sequencing of PCR products amplifying the sgRNA target regions. Of the 50 T_0_ shoots sequenced, 25 were derived from protoplasts transfected with the RNP-sgRNA1 complex, and 25 from the RNP-sgRNA2 complex. Nine sgRNA1 and seven sgRNA2 mutants were identified via Sanger sequencing of PCR product, indicating mutation efficiencies of 36% and 28%, respectively. The *in vitro* rooted plantlets of all mutants were grown in the biotron growth chambers until harvest for phenotypic evaluation and sinapine analysis in the seeds. Since the five mutant lines (B2, B5, B9, B15, and B20) from sgRNA1 in T_0_ were obtained first, they were chosen for further analysis. The selection of mutant lines for further evaluation in subsequent generations was based on sinapine content in comparison with WT.

**Figure 1:**
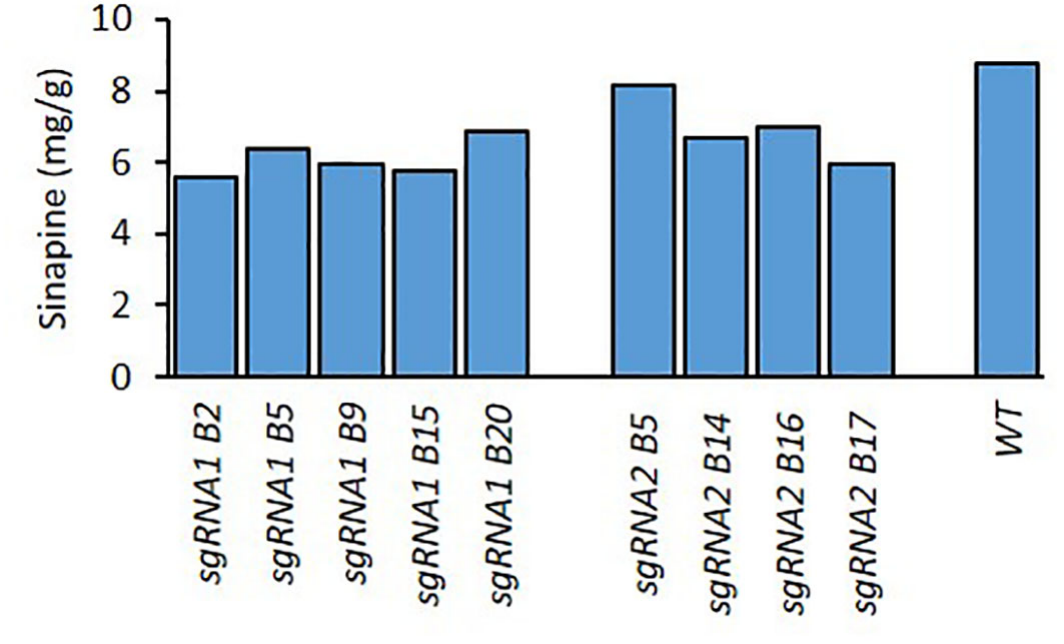
Sinapine content in the seeds of WT and *BnaX.SGT.a* mutants in the T_0_ generation of rapeseed.

**Figure 2:**
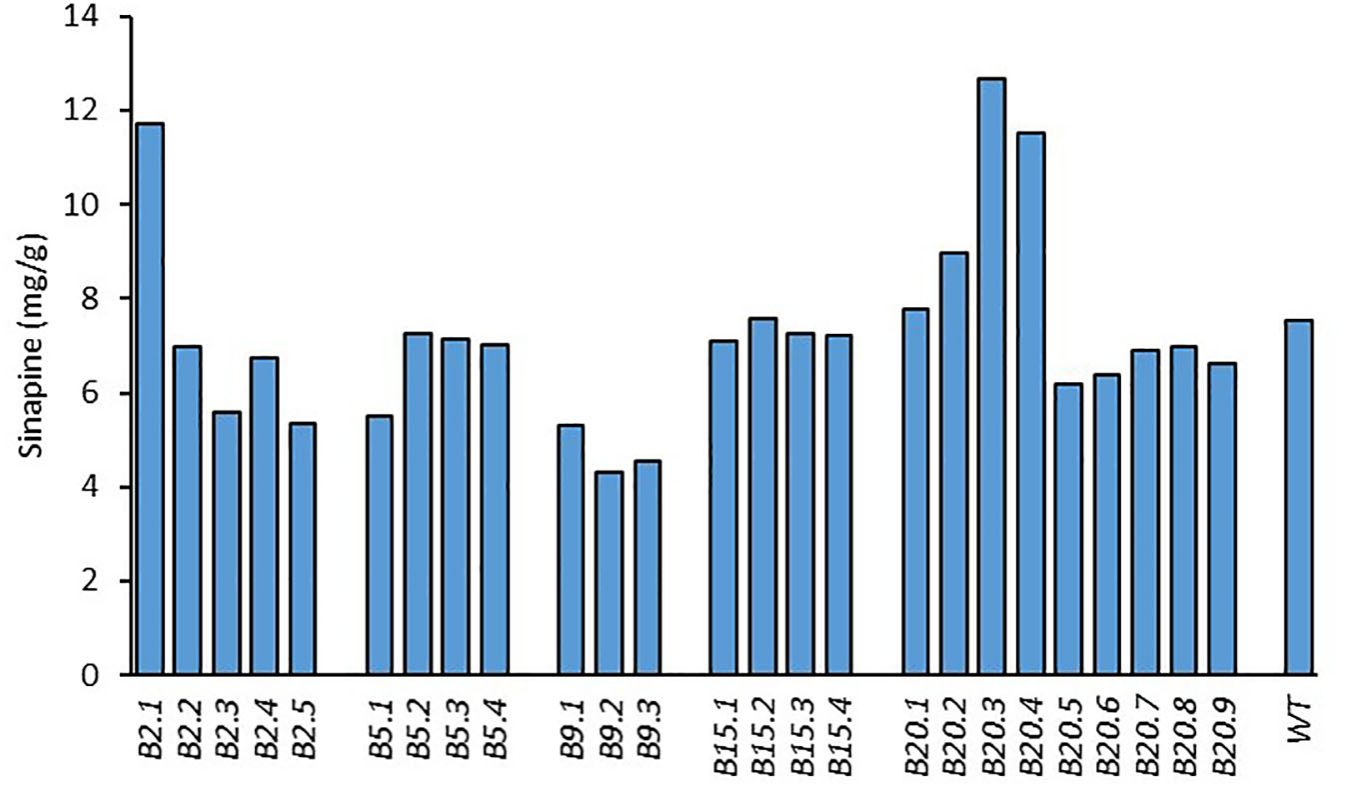
Sinapine content in the seeds of *BnaX.SGT.a* mutant lines in the T_1_ generation and WT of rapeseed.

For the T_1_ generation, at least 5 seeds from each of the 5 T_0_ mutant lines (**Figure 1**) were planted and grown in the biotron in order to confirm the stable inheritance of mutations. All plants were harvested, and their seeds were analysed for sinapine content (**Figure 2**). The 5 T_1_ mutant lines (B2.3, B2.5, B5.1, B9.2 and B9.3) with the lowest sinapine levels were selected, and 12 seeds from each line were grown in the biotron in the T_2_ generation for phenotypic and genotypic analysis for individual plants.

### Genotyping by amplicon sequencing

**Figure 3:**
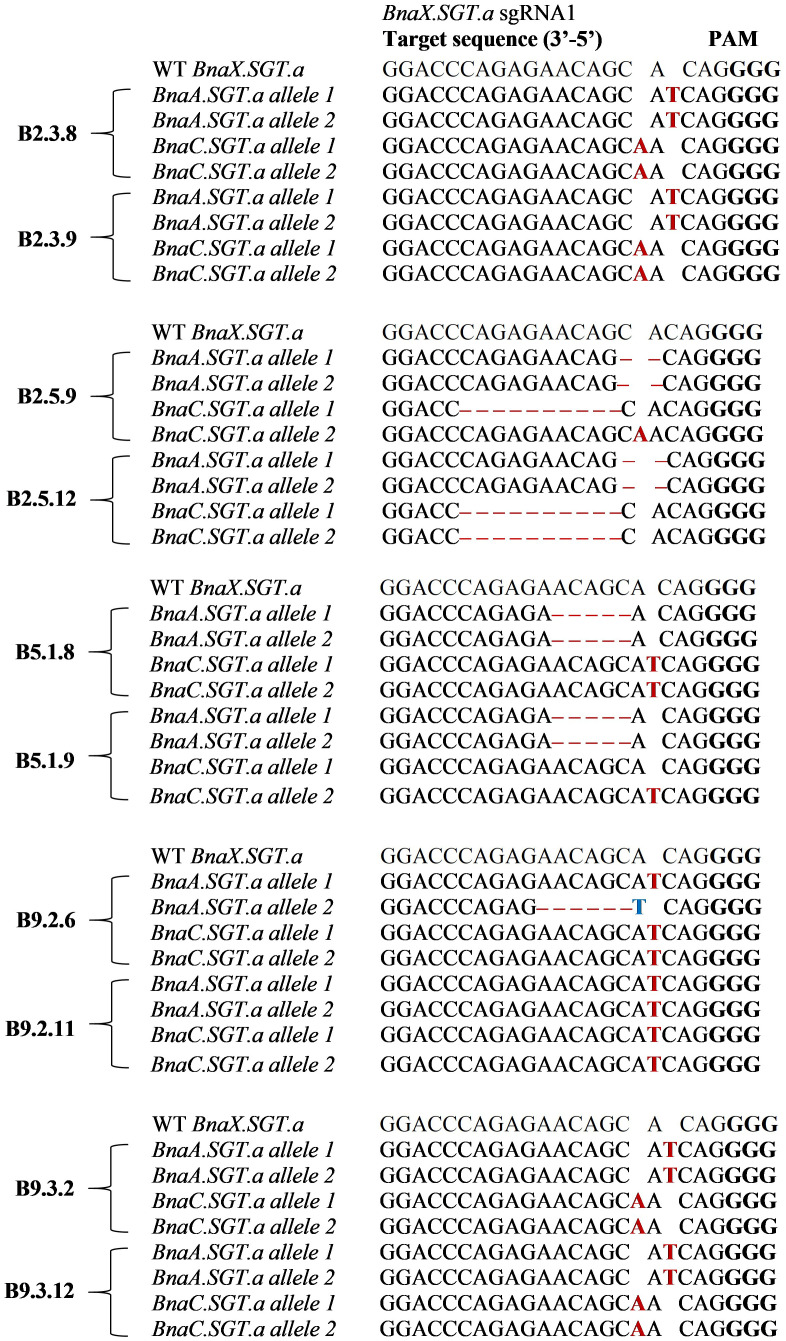
Types of mutations in the *BnaX.SGT.a* genes detected in the T_2_ mutants in comparison with WT of rapeseed, determined by amplicon sequencing. Mutations are indicated by red letters (insertions), ‘–’ (deletions), or blue letters (substitutions). PAM sites are highlighted in bold letters.

Two out of the twelve T_2_ plants from each line were genotyped using amplicon sequencing to confirm the types of mutations. The results showed that all plants were mutated in both alleles of both paralogues of *BnaX.SGT.a* (double mutants), apart from B5.1.9, which maintained one wild type allele (**Figure 3**).

A variety of mutations were induced by the CRISPR RNP editing, ranging from 1 bp insertions, to 10 bp deletions. All of the mutations caused a frameshift, apart from that of B9.2.6 *BnaA.SGT.a* allele 2, which had an in-frame nonsense mutation (**Figure 3**). All mutations led to premature stop codons, disrupting the predicted active site, homodimer interface, and TDP-binding site on conserved domain GT1_Gtf-like domain of the gene.

### Sinapine content

**Figure 4:**
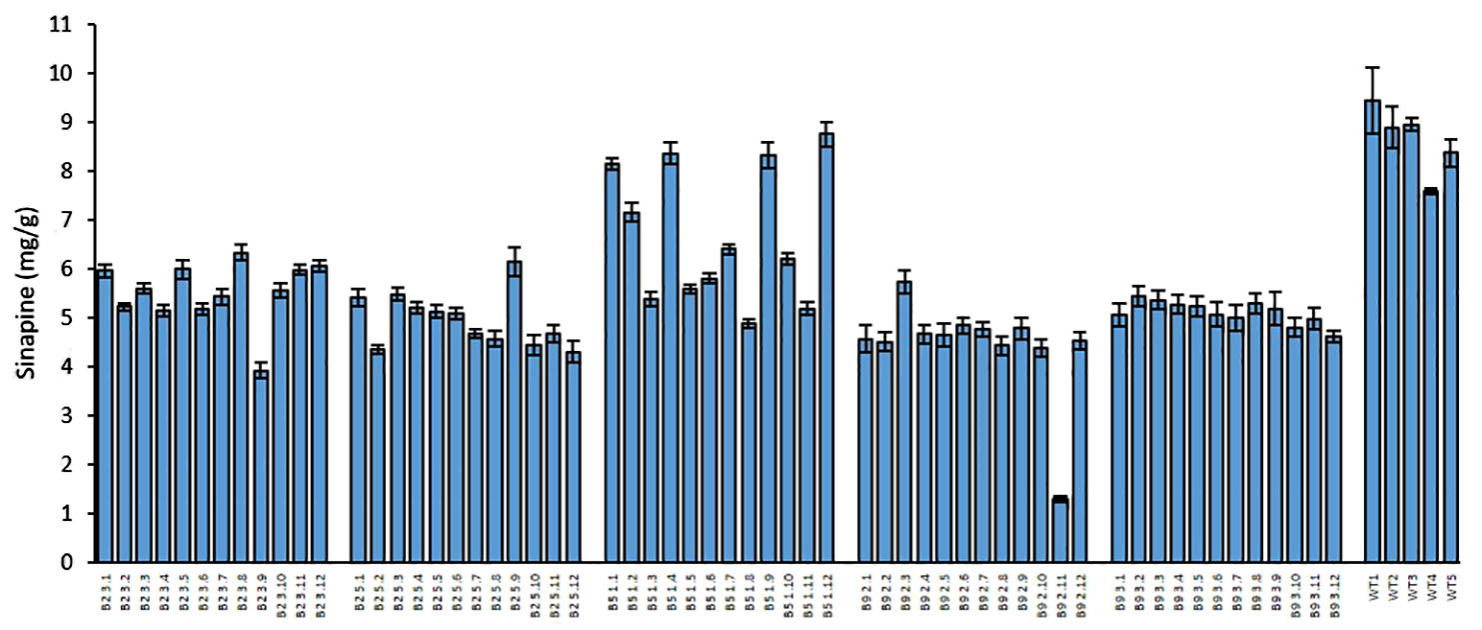
Sinapine content in the seeds of each individual plant in the T_2_ generation and WT of rapeseed. Error bars represent ± SD (n=3).

**Figure 5:**
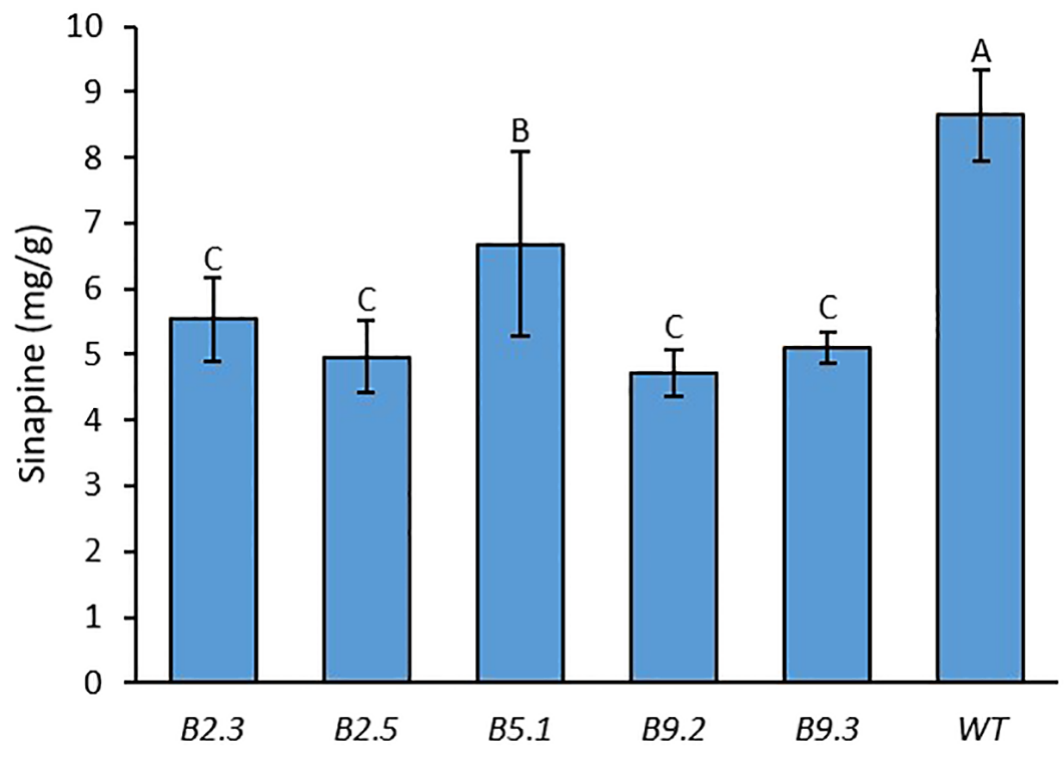
Average sinapine content of the seeds of all plants from each *BnaX.SGT.a* mutant line in the T_2_ generation in comparison with WT of 4 plants of rapeseed. Different letters above the bars represent significant differences at *p*<0.05. Error bars represent *±* SD.

We measured the sinapine content in mature seeds of the mutants and WT in each plant of all generations. As shown in **Figures 1**, **2** and **4**, an obvious reduction in sinapine content was detected in the majority of the lines analysed as early as the T_0_ generation (**Figure 1**). This reduction in sinapine content persisted in T_1_ (**Figure 2**), indicating stable inheritance of mutations. In the T_2_ generation, all mutant lines were shown to have significantly lower sinapine than WT (**Figures 4**, **5**), suggesting that homozygous lines were obtained. When comparing the efficacy of sgRNA1 and sgRNA2 in reducing sinapine content in T_0_ seeds, no significant difference in sinapine content was found between the two mutation sites.

The average sinapine content across all T_2_ double mutants was 5.08 mg/g, compared to 8.64 mg/g in the WT plants, reflecting a 41% reduction in the mutants. Among the T_2_ double mutants, line B9.2 exhibited the lowest average sinapine content at 4.72 mg/g, corresponding to a 45% reduction (**Figure 5**). The individual plant with the lowest sinapine content was B2.3.9, measured at 3.92 mg/g, representing a 49% reduction (**Figure 4**).

### Phenotypic observation

**Figure 6:**
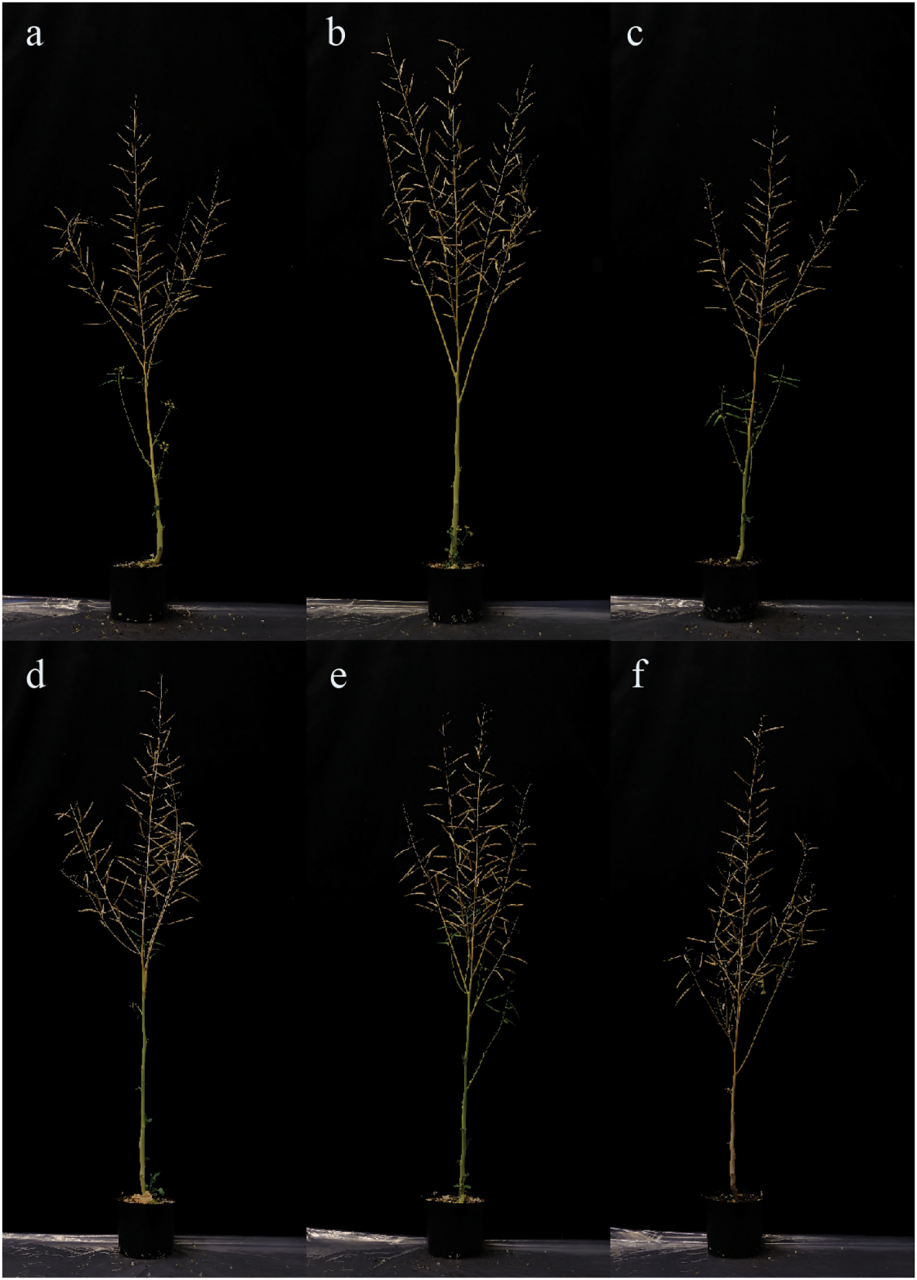
Photographs taken of *BnaX.SGT.a* mutants in the T_2_ generation and WT of rapeseed at harvest: **(A)** B2.3.8, **(B)** B2.5.9, **(C)** B5.1.8, **(D)** B9.2.6, **(E)** B9.3.2, **(F)** WT.

**Figure 7:**
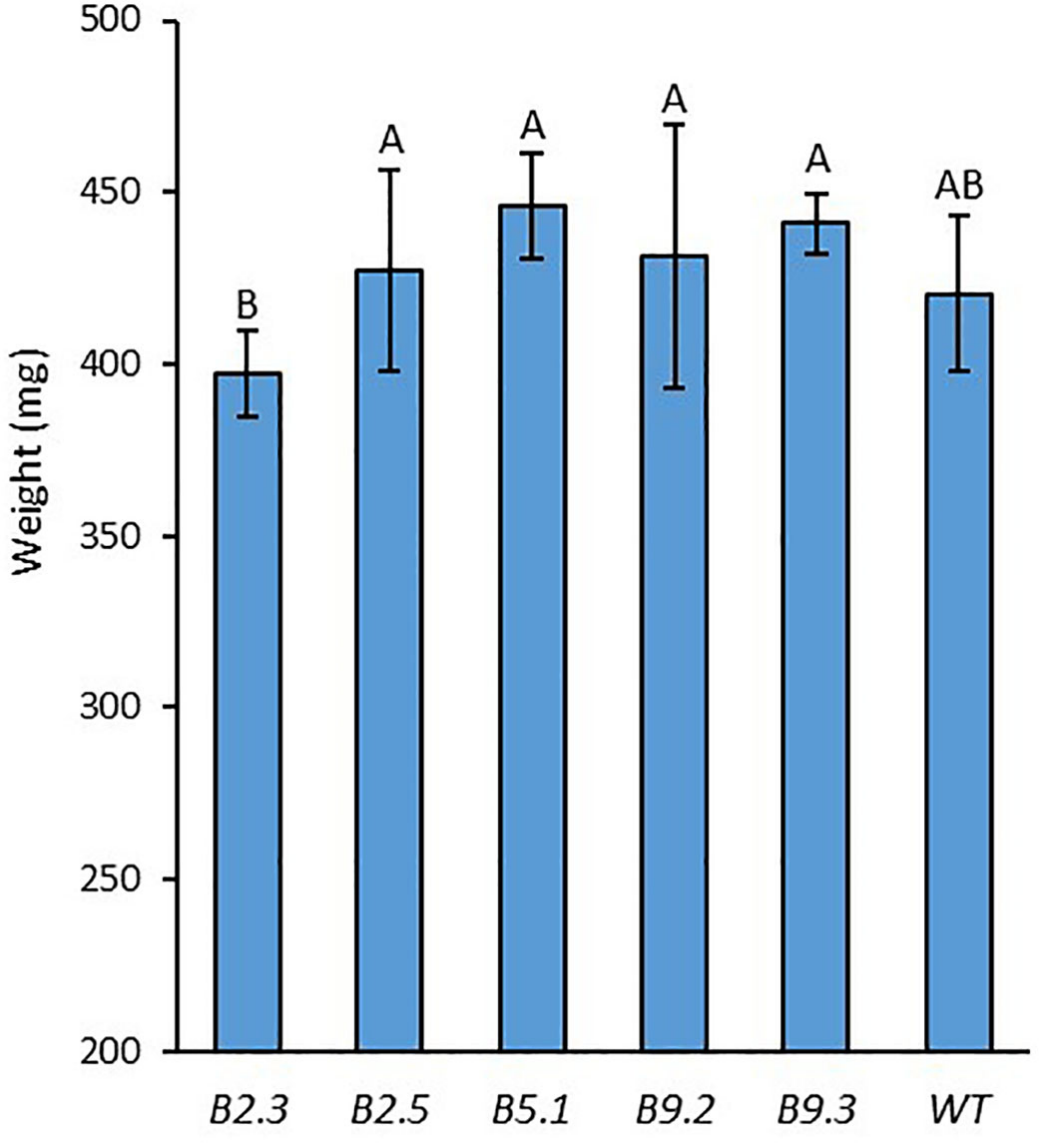
100-seed weight of *BnaX.SGT.a* mutants in the T_2_ generation and WT of rapeseed. Different letters above the bars represent significant differences at *p*<0.05. Error bars represent ± SD.

All of the homozygous T2 plants had visually normal growth and morphology (**Figure 6**). Fertility and flowering time did not visually differ from those of WT. The 100-seed weight result showed no significant difference between the mutant lines and WT, while the line B.2.3 showed a significantly lower seed weight than the other mutant lines (**Figure 7**).

## Discussion

The present study demonstrated the possibility of creating transgene free, low sinapine, mutants of rapeseed by knocking out the sinapine biosynthesis gene *SGT* using a protoplast-based CRISPR RNP approach. The success of this was made possible by a highly efficient protoplast protocol for rapeseed, which was developed in our lab (Li et al., 2021). In contrast to CRISPR mutagenesis via *Agrobacterium*-transformation, this approach enables a rapid production of transgene-free mutants with improved traits without the need for backcrossing to eliminate transgenic DNA. This distinction can be significant with regard to regulatory scrutiny and public perception of crops generated using NGTs. The benefits and low risks associated with transgene-free gene editing are gaining recognition, leading to growing acceptance worldwide (Dionglay, 2024). This trend is reflected in the European Union’s evolving stance regarding NGTs in plant breeding, with recent proposals aiming to relax regulations for gene-edited crops. Such regulatory adjustments signal a promising future for the broader adoption and use of NGTs in agriculture *(* European Parliament, 2024 *)*.

Sequencing of *Bna.SGT* in cv. Kumily showed that four paralogues of the gene exist, as reported by Mittasch et al. (2010). *BnaX.SGT.a* is the main paralogue expressed in rapeseed, and show increased expression during seed maturation. On the other hand the paralogue *BnaX.SGT.b* is only expressed at levels similar to *BnaX.SGT.a* in flowers, but has minimal expression in other tissues (Mittasch et al., 2010). We designed sgRNAs to target *BnaX.SGT.a* due to its predominant role in sinapine accumulation in the seeds of rapeseed, as was done in other studies (Hüsken et al., 2005; Wolfram et al., 2010; Emrani et al., 2015).

In the present study, we achieved an average editing efficiency of 36% and 28% for the sgRNA1 and sgRNA2, respectively. These mutation efficiencies are clearly higher than the DNA-vector induced mutation efficiency in rapeseed, in which 18% of mutation efficiency was obtained from our earlier studies using the same protoplast regeneration method (Li et al., 2021).

We observed a relatively uniform reduction in sinapine levels across the double homozygous lines, indicating that all plants within each line carry functionally similar loss-of-function mutations. In contrast, in the B5.1 line, some individuals showed a significant reduction in sinapine levels, while others had levels comparable to those in the WT. Sequencing results confirmed that this line is segregating: individuals with no reduction in sinapine levels carried knockout mutations in three of the four *BnaX.SGT.a* alleles, with one allele remaining unmutated, while those with significantly reduced sinapine levels had nonsense mutations in all alleles. This suggests that all four *BnaX.SGT.a* alleles need to be mutated to achieve a significant reduction in sinapine content. The maintenance of the WT sinapine phenotype when only one functional allele remains can potentially be sustained through compensatory mechanisms where the remaining functional allele is upregulated via a feedback loop, or through haplosufficiency where a single functional allele is sufficient for normal sinapine synthesis.

Conventional biotechnological approaches have previously been employed to silence *SGT* gene to reduce the seed sinapine level of rapeseed. Hüsken et al. (2005) used RNA interference (RNAi) to downregulate *SGT* expression and achieved a sinapine content of 2.7 mg/g, a 72% reduction compared to WT. Emrani et al. (2015) utilized EMS mutagenesis to create *SGT* knockouts, reaching 3.3 mg/g, a 57% reduction in comparison to WT. In the present study, we attained a sinapine content of 3.9 mg/g, a 49% reduction in comparison to WT.

The mutants developed by Hüsken et al. (2005) are classified as GMOs, while those generated by Emrani et al. (2015) through TILLING exhibited unintended phenotypic effects, such as severe changes in leaf morphology, shifts in flowering time, and reduced fertility and seed production. In contrast, the CRISPR-Cas9 mutants produced in the current study via protoplast-based CRISPR RNP editing showed no adverse effects on growth or development under biotron conditions, indicating that our approach is a promising tool for efficient and precise crop improvement without transgene integration, and without unintended off-target effects.

In gene knockout studies, a major concern is the potential for unintended effects arising from the removing of a gene that may be involved in other biochemical pathways or affect the plant’s growth and overall health. We did not visually observe any negative phenotypic changes in the *SGT* knockout lines, which is in concurrence with previous studies that have evaluated the effect of silencing *SGT* (Hüsken et al., 2005; Wolfram et al., 2010; Emrani et al., 2015; Hettwer et al., 2016). Hettwer et al. (2016) concluded that suppression of *SGT* resulted in reduced sinapine and sinapate ester accumulation, with no adverse effect on seed germination, seedling development, or response to UV-B radiation (Hettwer et al., 2016). Additionally, no negative effects were observed on key agricultural traits such as oil content, fatty acid composition, or protein content in the seeds (Hüsken et al., 2005).

In this study the CRISPR-edited mutants showed significant reduction in sinapine levels, further confirming the importance of *BnaX.SGT.a* in sinapine biosynthesis in rapeseed. However, the sinapine levels in the mutants did not reach levels as low as those reported in previous studies on *BnaX.SGT.a* (Hüsken et al., 2005; Wolfram et al., 2010; Emrani et al., 2015). This discrepancy may be attributed to mutations occurring in different positions in the gene, leading to alternative protein variants that influence sinapine biosynthesis in different ways. Another possible explanation for the lesser reduction in sinapine levels is that we screened fewer lines for sinapine content, which may have led us to overlook low-sinapine variants. Significant variability exists among different mutant lines, and prior studies have screened a larger number of mutants, potentially enabling the identification of variants with lower sinapine levels (Hüsken et al., 2005; Emrani et al., 2015).

To further reduce sinapine levels using our protoplast-based CRISPR RNP editing method, several strategies can be considered. One approach is to screen a larger number of mutants to identify those with lower sinapine content. Another approach would be to design multiple sgRNAs to simultaneously target all four *SGT* loci may be beneficial, as the less-expressed *BnaX.SGT.b* genes, which were not targeted in the current study, could be compensating for the inactivity of the more highly expressed *BnaX.SGT.a* genes.

The most promising strategy for achieving significantly lower sinapine levels may be to target more than one gene simultaneously. For instance, the highest reduction in sinapine has been achieved by concurrently silencing two genes, *FAH* and *SCT*, through RNAi, resulting in a 90% reduction in seed sinapine levels (Bhinu et al., 2009). In the present study we achieved up to a 49% reduction in sinapine content, reaching 3.9 mg/g. However, this level remains higher than the 2 mg/g target recommended as the major breeding goal (Harloff et al., 2012).

In conclusion, we successfully generated transgene-free rapeseed mutants with significantly reduced sinapine levels by knocking out *SGT* using our protoplast-based CRISPR RNP editing approach. This demonstrates the feasibility of rapidly creating new plant varieties with stable, heritable traits that remain transgene-free throughout all stages of production. The approach is notably faster and more cost-effective than conventional breeding methods. Additionally, CRISPR-generated mutants are likely to encounter less stringent regulatory oversight than conventional GMOs in the near future, providing a promising strategy for efficiently reducing sinapine and other anti-nutritional factors in crops.

## Data Availability

The raw data supporting the conclusions of this article will be made available by the authors, without undue reservation.
